# Efficacy of Different Precooling Agents and Topical Anesthetics on the Pain Perception during Intraoral Injection: A Comparative Clinical Study

**DOI:** 10.5005/jp-journals-10005-1296

**Published:** 2015-08-11

**Authors:** Garima Lathwal, Inder Kumar Pandit, Neeraj Gugnani, Monika Gupta

**Affiliations:** Senior Lecturer, Department of Pedodontics and Preventive Dentistry, Yamuna Institute of Dental Sciences and Research, Yamuna Nagar Haryana, India; Professor and Head, Department of Pedodontics and Preventive Dentistry, DAV Centenary Dental College and Hospital, Yamuna Nagar Haryana, India; Professor, Department of Pedodontics and Preventive Dentistry, DAV Centenary Dental College and Hospital, Yamuna Nagar Haryana, India; Professor, Department of Pedodontics and Preventive Dentistry, DAV Centenary Dental College and Hospital, Yamuna Nagar Haryana, India

**Keywords:** Local anesthesia, Precooling agents, Topical anesthesia.

## Abstract

Topical anesthesia is widely advocated in pediatric dentistry practice to reduce pain and anxiety produced by administration of local anesthesia. Cryoanesthesia to lessen the injection pain has also been reported to be promising. However, sparse literature reports exist regarding clinical efficacy of these agents.

**Aim:** The purpose of this study was to compare the efficacy of the refrigerant (1,1,1,3,3-pentafluoropropane/1,1,1,2-tetrafluo-roethane), benzocaine and ice on the pain perception during intraoral injection using visual analog scale (VAS) and sound, eye, motor (SEM) scale.

**Study design:** In this Spit-mouth design study, a total of 160 patients between the age group of 5 and 8 years were selected and were randomly divided into two equal groups having 80 patients in each group.

**Results:** Ice cone has shown lower mean scores (p < 0.001) as compared to benzocaine and refrigerant whereas no significant difference was observed between refrigerant and benzocaine (p > 0.05) on both the scales.

**Conclusion:** Ice cone had shown significantly higher efficacy as compared to benzocaine and refrigerant.

**How to cite this article:** Lathwal G, Pandit IK, Gugnani N, Gupta M. Efficacy of Different Precooling Agents and Topical Anesthetics on the Pain Perception during Intraoral Injection: A Comparative Clinical Study. Int J Clin Pediatr Dent 2015;8(2):119-122.

## INTRODUCTION

Local anesthesia is required in any dental practice including pedodontics to alleviate the pain of dental procedures like extractions, pulpotomies, root canal treatments/ pulpectomies, drainage of abscesses and minor oral surgical procedures. However, the irony of the situation is that local anesthetics which are the most effective drugs for the prevention and management of pain^1^ are themselves associated with pain and this pain gets further aggravated due to the fear and anxiety caused by the sight of the needle and has been referred to as needle phobia or blenophobia.^2^

The most widely advocated technique to minimize the pain of local anesthesia is the use of topical anesthetic agent before injection. Benzocaine due to its prolonged effect and acceptable taste is the most popular topical anesthetic agent used in dentistry. Cryoanesthesia is the application of cold to a localized part of the body in order to block the local nerve conduction of painful impulses. It may be induced either by the use of refrigerant sprays or with the use of ice. The focal application of ice before and sometimes after painful procedures has been practiced for thousands of years and was one of the first source of local anesthesia and analgesia.

A new vapocoolant spray Gebauer’s pain ease was introduced and approved by US Food and Drug Administration in the year 2004. Gebauer’s Pain ease is a proprietary blend of 1,1,1,3,3-pentafluoropropane and 1,1,1,2-tetrafluoroethane and is non-flammable and non-ozone depleting spray.

Thus, with the objective of reducing unpleasant experience of needle prick the present clinical study aimed to evaluate and compare the efficacy of different pre-cooling agents and topical anesthetics on the pain perception during intraoral injection.

## MATERIALS AND METHODS (FLOW CHART 1)

A total of 160 healthy children with no history of systemic diseases (ASA Grade I status) and without any allergic history to local anesthesia in the age group of 5 to 8 years were selected from the outpatient clinics in the department of pedodontics and preventive dentistry at JN Kapoor DAV Centenary Dental College and Hospital, Yamuna Nagar, Haryana.

**Flow Chart 1:**
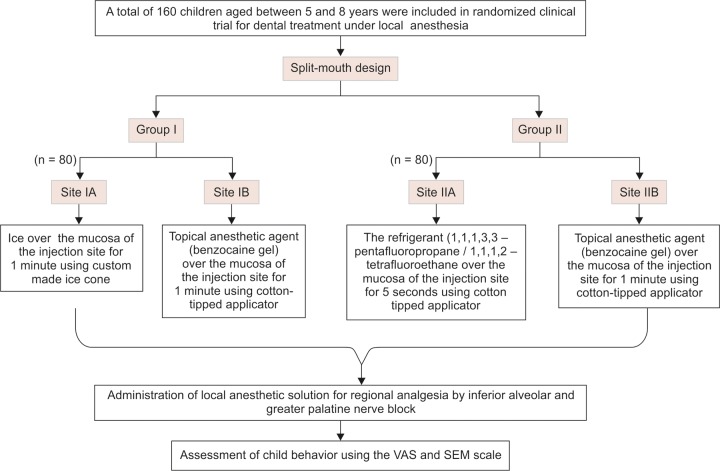
Methodology

It was a split-mouth parallel randomized study, so patients requiring bilateral local anesthetic block (either bilateral inferior alveolar nerve block or bilateral greater palatine nerve block) for any of the dental procedures in either of the jaws were recruited. Selected patients were then randomly divided into two equal groups (groups I and II) having 80 patients in each group (Table 1). Randomization was done using the computer generated random equal numbers of blinded packages containing either of the group code. Blinded packages were prepared by the nonclinical staff according to the generated random chart and were available to the investigator only after the child was recruited for the study.

Once the group was selected, the sites for the application of topical anesthetic agents and precooling agents were selected randomly by coin toss method and the selected sites were then named as site IA, IB and IIA, IIB for their respective groups.

**Table 1:** **Table 1:** Division of samples

*Group number*		*Sample site*		*Size number*		*Material used*	
I		80		IA		Ice Cone	
				IB		Benzocaine gel	
II		80		IIA		Refrigerant IIB	
						Benzocaine gel	

In group I, site IA was treated with custom made ice cone and site IB was treated with benzocaine gel, both for 1 minute. Whereas, in group II, site IIA was treated with refrigerant for 5 seconds and site IIB was treated with benzocaine gel for 1 minute. Thus, benzocaine gel was used in both the groups of the study and therefore, acted as control (site IB and site IIB). The procedures were carried out after wiping the mucosa in relation to the area of needle penetration free of saliva and after maintenance of isolation with the help of cotton rolls and suction tips.

**Fig. 1:**
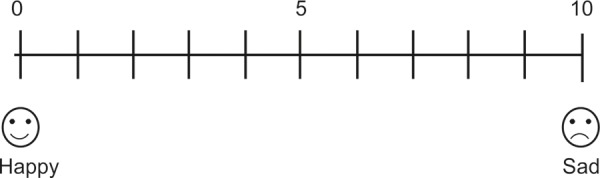
Visual analog scale for the assessment of child’s pain

In all sites of both the groups after the application of anesthetic agent, administration of local anesthetic injection with a 1 inch, 25 mm gauge needle was done. During the insertion of needle, the patient’s behaviour was evaluated for pain perception using sound, eye, motor (SEM) scale (Table 2) and visual analog scale (VAS) by the operator (Fig. 1).

The statistical analysis was done using Statistical Package for Social Sciences (SPSS) Version 15.0 Statistical Analysis Software. The values were represented in mean ± SD.

**Table 2:** **Table 2:** Sound, eye, motor scale for the assessment of child’s behavior

*Parameter*		*Comfort 1*		*Mild discomfort 2*		*Moderate discomfort 3*		*Severe discomfort 4*	
Sound		No sound		Non-specific sound		Verbal complaint, louder sound		Verbal complaint, shouting, crying	
Eye		No sign		Dilated eyes without tears (anxiety sign)		Tears, sudden eye movements		Crying, tears covering the face	
Motor		Relaxed body and hand status		Muscular contraction, contraction of hands		Sudden body and hand movements		Hand movement for defence, turning the head to opposite side	

## OBSERVATIONS AND RESULTS

**Graph 1:**
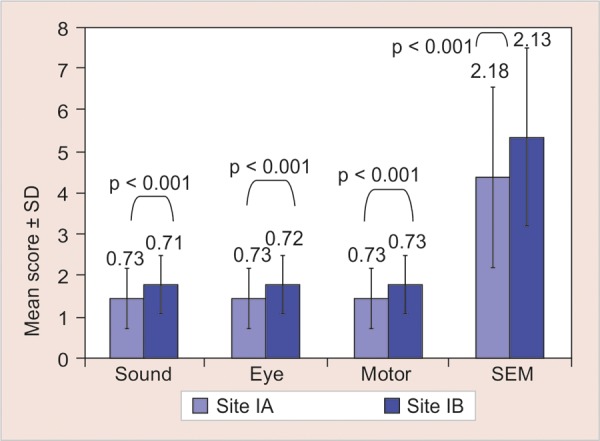
Intragroup comparison of mean scores of SEM scale between test site (IA) and control site (IB) in group I of the study

Graph 1 shows that the mean value of Ice cone were significantly lower (p < 0.001) when intragroup comparison was made with benzocaine on SEM scale using Wilcoxon Signed rank test. No significant difference (p > 0.05) was observed on comparing refrigerant with benzocaine.

**Graph 2:**
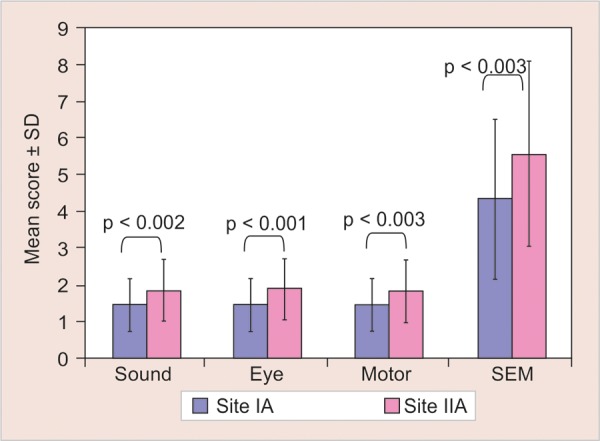
Intergroup comparison of SEM scores between test sites (site IA and IIA) of the study using Mann-Whitney U test

**Graph 3:**
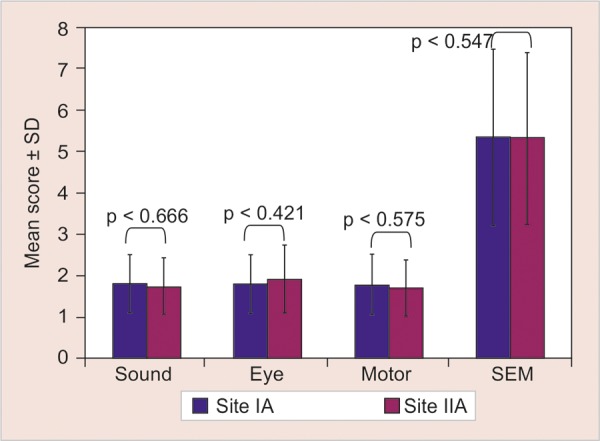
Intergroup comparison of SEM scores between control sites (site IB and IIB) of the study using Mann-Whitney U test

Graph 2 shows significant difference between the mean scores of Ice and refrigerant (p < 0.001) on SEM scale and Graph 3 shows nonsignificant difference between control sites (p > 0.05) on SEM scale when comparisons were made using Mann-Whitney U test.

**Graph 4:**
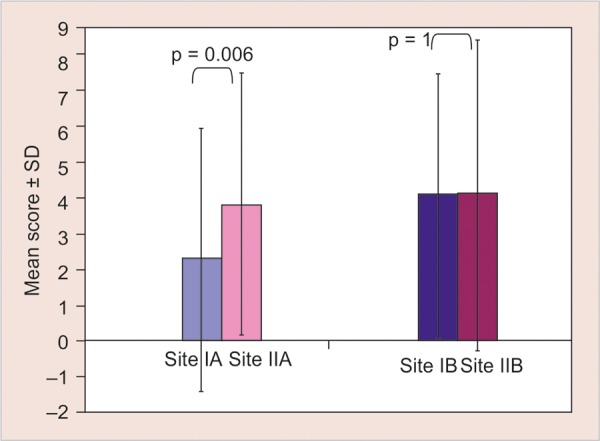
Intergroup comparison of VAS scores for pain between test sites (site IA and IIA) and control sites (site IB and IIB) of the study using Mann-Whitney U test

Graph 4 shows significant difference (p = 0.006) when comparisons of Ice cone and refrigerant were evaluated on VAS scale using Mann-Whitney U test. No difference was found between the two control sites (benzocaine) of both the groups (NS; p = 1).

## DISCUSSION

Pain control is a challenging task in clinical pediatric dentistry. Conventional pain control techniques, however, deal with only one aspect of pain control, i.e. pharmacological/sensory, the psychologic component is often left unresolved. This is especially true of the pediatric population where the fear of needle is a major deterrent to quality dental care. It is ironical that to eliminate pain we must momentarily create a painful stimulus.

Cryoanesthesia may be induced either by the use of refrigerant sprays or with the use of ice. The chief benefit of cryoanesthesia is that, it acts on all the cells of the part and not just on the nerve cells as other topical anesthetics and analgesics do, thereby, producing an immediate anesthesia.^3^ The anesthesia produced by cryoanesthesia is of very short duration (2 to 5 second) but is sufficient enough to reduce the discomfort caused by the insertion of a needle.^4^

In the study, the patient’s behavior was evaluated for pain perception using SEM scale and VAS by the operator. Sound, eye, motor scale takes into account the SEM component of patients response to stimulation and was used because it enables the assessment of the relationship between pain and the reactions which the sensation of pain generates in the patient’s eyes, bodily movements and verbal expressions of discomfort and also it is able to record the degree of intensity of the sensation of pain. The other scale used in the study was VAS which is a form of cross-modality matching in which the length of a line is adjusted to match the strength of a perception.^5^ In this, the child was asked to rate the discomfort of the injection on a 10 cm scale where 0 represented a happy child with no pain at one end and 10 represented a crying child with extreme pain at the other end. Median value 5 represented sad child with moderate amount of pain. The median value was taken in order to get more sensitive and accurate representation of pain intensity.

The increased efficacy of ice (site IA) was probably due to its increased contact time with the tissues as compared to the refrigerant (site IIA). Though free nerve endings abound and terminate in all layers of mucosa, yet, they have different speed of conduction^6^ and this increased contact time of ice might have resulted in slowing the velocity of nerve impulse induction of almost all the types of nerve fibers. Moreover, the mechanism of action of refrigerant is also limited to the surface of application site and it works by creating an instant cooling effect while evaporating from the mucous membrane. This mechanism creates almost immediate onset of action, however, along with immediate onset, refrigerant also has the disadvantage of brief duration of action^7^, which might be responsible for its low efficacy as compared to ice cone. These results are in agreement to the study in which 1 minute ice cube application was found to be significantly more effective than the 5 seconds application of vapocoolant spray.^8^

The advantage of this technique is that it comfortable, safe and physiologically effective. Moreover, ice is inexpensive and readily available everywhere in India and is a material which is familiar to the patient’s, thereby, is less likely to induce anxiety and subjective fear. Future studies can expand on our research and examine other materials and techniques to compare their effectiveness as a preinjection anesthetic with that of ice cone.

## CONCLUSION

It was concluded from the study that all the three surface anesthetic agents were effective in significantly reducing the pain perception associated with intraoral injection. Ice cone showed significantly higher efficacy as compared to Benzocaine gel and Refrigerant.

## References

[1] Aminabadi N Asl, Farahani RMZ (2009). The effect of Pre-cooling the Injection site on pediatric pain perception during administration of local anesthesia.. J Contemp Dent Pract.

[2] Kosaraju A, Vandewalle KS (2009). A Comparison of a refrigerant and a topical anesthetic gel as preinjection anesthetics.. J Am Dent Assoc.

[3] Atkinson RS, Rushman GB, Alfred LJ (1987). A synopsis of anaesthesia. Textbook of Anaesthesia, Wright Publication.

[4] Michael JA (1993). New Developments in local anesthesia. Textbook of Surgical Dermatology: Advances in Current Practice, St Louis Mosby Publication.

[5] Patricia AM (1987). An assessment of Children’s pain: a review of behavioral, physiological and direct scaling techniques.. Pain.

[6] Harbert H (1989). Topical ice: a precursor to palatal injections.. J Endod.

[7] Steven JW (2010). Topical local anesthetics.. Textbook of the essence of analgesia and analgesics.

[8] Yoon WY, Chung SP, Lee HS, Park YS (2008). Analgesic pretreatment for antibiotic skin test: vapocoolant spray vs ice cube.. Am J Emerg Med.

